# Acylglycerols of Myristic Acid as New Candidates for Effective Stigmasterol Delivery—Design, Synthesis, and the Influence on Physicochemical Properties of Liposomes

**DOI:** 10.3390/molecules27113406

**Published:** 2022-05-25

**Authors:** Witold Gładkowski, Aleksandra Włoch, Hanna Pruchnik, Anna Chojnacka, Aleksandra Grudniewska, Agnieszka Wysota, Anna Dunal, Daniel Rubiano Castro, Magdalena Rudzińska

**Affiliations:** 1 Department of Food Chemistry and Biocatalysis, Wrocław University of Environmental and Life Sciences, Norwida 25, 50-375 Wrocław, Poland; anna.chojnacka@upwr.edu.pl (A.C.); aleksandra.grudniewska@upwr.edu.pl (A.G.); 109799@student.upwr.edu.pl (A.W.); 2 Department of Physics and Biophysics, Wrocław University of Environmental and Life Sciences, Norwida 25, 50-375 Wrocław, Poland; hanna.pruchnik@upwr.edu.pl; 3 Facultat de Biologia, Universitat de Barcelona, Avinguda de Diagonal 643, 08007 Barcelona, Spain; drubiaca7@alumnes.ub.edu; 4 Faculty of Food Science and Nutrition, Poznań University of Life Sciences, 60-637 Poznań, Poland; magdalena.rudzinska@up.poznan.pl

**Keywords:** new lipid nanocarriers, liposomes, acylglycerols, stigmasterol, Steglich esterification, physicochemical properties

## Abstract

New carriers of phytosterols; acylglycerols containing natural myristic acid at *sn*-1 and *sn*-3 positions and stigmasterol residue linked to *sn*-2 position by carbonate and succinate linker have been designed and synthesized in three-step synthesis from dihydroxyacetone (DHA). The synthetic pathway involved Steglich esterification of DHA with myristic acid; reduction of carbonyl group of 1,3-dimyristoylpropanone and esterification of 1,3-dimyristoylglicerol with stigmasterol chloroformate or stigmasterol hemisuccinate. The structure of the obtained hybrids was established by the spectroscopic methods (NMR; IR; HRMS). Obtained hybrid molecules were used to form new liposomes in the mixture with model phospholipid and their effect on their physicochemical properties was determined, including the polarity, fluidity, and main phase transition of liposomes using differential scanning calorimetry and fluorimetric methods. The results confirm the significant effect of both stigmasterol-containing acylglycerols on the hydrophilic and hydrophobic region of liposome membranes. They significantly increase the order in the polar heads of the lipid bilayer and increase the rigidity in the hydrophobic region. Moreover, the presence of both acylglycerols in the membranes shifts the temperature of the main phase transition towards higher temperatures. Our results indicate stabilization of the bilayer over a wide temperature range (above and below the phase transition temperature), which in addition to the beneficial effects of phytosterols on human health makes them more attractive components of novel lipid nanocarriers compared to cholesterol.

## 1. Introduction

Phytosterols are structural and functional analogues of cholesterol, synthesized by plants. These compounds are essential components of cell membranes that regulate their physicochemical properties. The most frequently referred plant sterols are 4-desmethyl sterols. Among them, one of the most common in the human diet is stigmasterol (stigmasta-5,22-dien-3β-ol) [1]. As other phytosterols, stigmasterol is known for its cholesterol-lowering properties [2,3]. Besides, many other health benefits of stigmasterol have been proved. It was shown to alleviate nonalcoholic fatty liver disease (NAFLD) via suppression of hepatic lipogenic gene expression and modulation of circulating ceramide levels [4]. By inhibition of several pro-inflammatory mediators involved in cartilage degradation, stigmasterol exhibits anti-osteoarthritic properties [5,6]. Stigmasterol is a promising compound for the prevention of neurodegenerative diseases, including Alzheimer’s disease, as it exerts the effect against oxidative stress-induced apoptosis via the antioxidative defense mechanism of hydrogen peroxide reduction [7]. Stigmasterol also has an antidiabetic effect by the prevention of defects induced by glucolipotoxicity in glucose-stimulated insulin secretion [8]. This phytosterol was also shown to exert anticancer effects on the human gastric cancer [9] and cholangiocarcinoma cells [10]. It can also find an application as an antibiotic adjuvant because of its synergistic effects with ampicillin against bacterial strains producing beta lactamase [11].

Due to the low oil solubility and bioavailability of free stigmasterol, it is worth focusing on increasing its supply to the target tissues by its conjugation with other hydrophobic molecules. In this aspect, we paid our attention to acylglycerols, which, due to their natural presence both in food and the human body, can be an effective lipid delivery system and have been successively used as carriers of different bioactive ingredients [12], including fatty acids [13,14], carotenoids and selena fatty acids [15], phenolic acids [16,17] or non-steroidal anti-inflammatory drugs [18,19]. 

Due to their structure, acylglycerols are also an excellent base for their use as the components of liposomes, which may increase their stability and efficiency to deliver stigmasterol to the human body. Lipids in which two molecules of stigmasterol are covalently linked via succinate linker to glycerophosphocholine were used previously as the components of liposome formulations of doxorubicin and amphotericin B [20,21]. To the best of our knowledge, the use of hybrids of acylglycerol and phytosterols in the formation of liposomes has not been reported so far.

In our previous work, we synthesized distigmasterol-modified acylglycerols [22] containing palmitic or oleic acid. Now our attention has also been attracted by myristic acid (MA), exhibiting unique physiological properties compared to other saturated fatty acids. MA intake is positively correlated with the concentrations of cellular bioactive lipids including n-3 polyunsaturated fatty acids (*n*-3 PUFA) and ceramide. It is the result of the myristoylation of *N*-terminal glycine residues of some enzymes, like NADH-cytochrome b5 reductase and dihydroceramide Δ4-desaturase [23]. The myristoylation of the former accounts for the increased Δ6-desaturase activity, which is the crucial enzyme in the conversion of α-linolenic acid to eicosapentaenoic acid (EPA) and docosahexaenoic acid (DHA) [24]. Among different fatty acids, only MA exerts anxiolytic-like effects, comparable to diazepam, in studies using Wistar rats [25].

The aim of this work was a design and synthesis of structured acylglycerols containing myristic acid residues at *sn*-1 and *sn*-3 positions and the stigmasterol molecule attached to the *sn*-2 position. Additionally, the obtained hybrid molecules were used to form new liposomes in the mixture with model phospholipid. The physicochemical properties of newly obtained liposomes were determined, including fluidity of their membranes and their thermotropic parameters such as the temperature of the main phase transition and half width of the main peak. Understanding the physicochemical parameters of liposomes will allow us to determine their properties and their stability, which is crucial for their practical application in the food industry as food additives or as nutraceuticals. 

## 2. Results

### 2.1. Synthesis

**Scheme 1:**
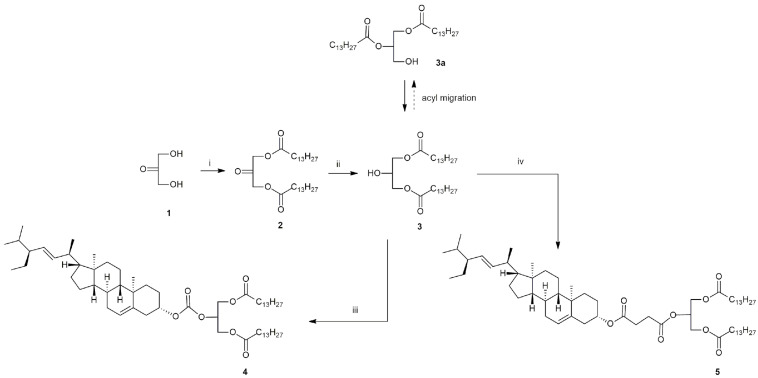
Synthesis of acylglycerols of myristic acid containing stigmasterol residue at *sn*-2 position. *Reagents and conditions:* (i) myristic acid, DCC, DMAP, CHCl_3_, r.t., 24 h (ii) NaBH_4_, THF, −14 °C, 30 min (iii) stigmasteryl chloroformate, DMAP, CHCl_3_, r.t., 48 h (iv) stigmasteryl hemisuccinate, DCC, DMAP, CHCl_3_, r.t., 24 h.

Target acylglycerols containing stigmasterol molecule (**4,5**) were obtained in three-step synthesis (Scheme 1). 

**Scheme 2:**
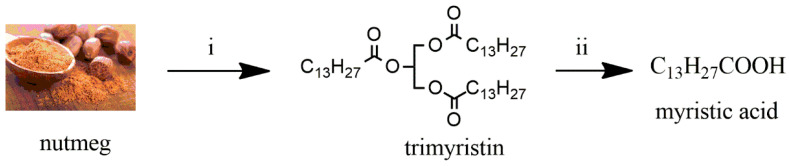
Production of myristic acid from nutmeg. Reagents and conditions: (i) hexane, reflux, 1 h, then crystallization from acetone (ii) NaOH, EtOH, reflux, 1.5 h, then HCl.

The starting substrates were commercially available dihydroxyacetone (DHA, **1**) and myristic acid of natural origin. The latter was obtained according to the known procedure as a result of the saponification of trimyristin isolated from nutmeg by Soxhlet extraction (Scheme 2). Following this methodology, 2.81 g of MA with 95% purity, which contained 5% of palmitic acid, was obtained. Purities of trimyristin and myristic acid were calculated based on their fatty acid composition determined by gas chromatography after standard derivatization of the samples into methyl esters.

In the first step of synthesis, Steglich esterification [26] was applied to esterify both hydroxy groups of DHA with MA and 1,3-dimyristoylpropanone (**2**) was obtained as the only product in 75% isolated yield. In the subsequent step the reduction of the carbonyl group of 1,3-dimyristoylpropanone (**2**) was necessary. The reaction was carried out in a THF ice bath using sodium borohydride and after 30 min the ketone **2** reacted completely, but surprisingly two products, **3** and **3a**, were detected in the reaction mixture on the TLC plate. After usual work-up of the reaction mixture, they were separated by flash chromatography. Careful spectroscopic analysis let us to identify the major, less polar compound (R*_f_* = 0.22), obtained in 56% yield, as the expected product of reduction, 1,3-dimyristoylglycerol (**3**) and the minor, more polar compound (R*_f_* = 0.16) as *rac*-1,2-dimyristoylglycerol (**3a**), obtained in 23% yield.

The key signals in the ^1^H NMR spectra, crucial for the determination of structure of diacylglycerols **3** and **3a,** were those observed for the diastereotopic protons of CH_2_-1 and CH_2_-3 of the glycerol skeleton. The symmetry of 1,3-dimyristoylglycerol (**3**) caused the mentioned protons to give two doublets at 4.13 and 4.18 ppm, each represented by two magnetically equivalent protons: one from CH_2_-1 group and one of CH_2_-3 group. In the molecule of 1,2-isomer **3a** four doublets of doublets were observed in total. Protons from the CH_2_-1 group gave two signals at a significantly lower field (4.23 and 4.32 ppm) than protons from the CH_2_-3 group (3.71 and 3.74 ppm). Observed difference in chemical shifts as well as magnetic nonequivalence of these protons was caused by the presence of an ester group at C-1 and OH group at C-3. This also resulted in the clear differentiation of signals from two carbonyl carbons between isomers **3** and **3a** on their ^13^C NMR spectra. In the case of isomer **3a** containing myristic acid esterified at *sn*-2 and *sn*-1 position, the separate signals at 173.46 and 173.84 ppm were observed, respectively. In the case of isomer **3**, in which two molecules of myristic acid were attached at *sn*-1 and *sn*-3 positions, only one signal from carbonyl carbons was detected at 173.95 ppm.

Formation of *rac*-1,2-dimirystoylglycerol (**3a**) during the reduction of ketone **2** by sodium borohydride was also observed by the Cockman et al. as the effect of migration of myristoyl residue from *sn*-1/*sn*-3 to *sn*-2 position of glycerol backbone in the product of reduction, 1,3-dimyristoyl glycerol (**3**) [27]. The authors detected the formation of **3a** by TLC and ^13^C NMR without the separation of the products mixture. In this work, both diacylglycerols **3** and **3a** after separation were fully characterized using spectroscopic methods to confirm the acyl migration, which is a common phenomenon in the synthesis of structured acylglycerols, including enzymatic and chemical reaction and often limits the reaction yield leading to the formation of by-products [28,29].

In the final step of synthesis, purified 1,3-dimyristoylglycerol (**3**) was conjugated with stigmasterol by carbonate linker in the reaction with stigmasteryl chloroformate as well as by succinate linker in the Steglich-type esterification with stigmasteryl hemisuccinate to afford 1,3-dimyristoyl-2-stigmasterylcarbonoylglycerol (**4**) and 1,3-dimyristoyl-2-stigmasterylsuccinoylglycerol (**5**), respectively. Their molecular masses were determined by HRMS analysis, and their structures were confirmed by NMR measurements. The signals from the stigmasterol part were assigned to corresponding protons and carbons based on the previous literature data [22,30]. The presence of myristic acid residues was confirmed by triplets at 0.88 ppm from terminal methyl groups of hydrocarbonate chains, triplets in the range 2.21–2.32 ppm from CH_2_ group in α-position as well as the multiplets from methylene groups of myristic acid in the range 1.22–1.33 ppm. On the ^13^C NMR spectrum of acylglycerol **4**, the signal from carbon of carbonate linker was detected at 153.73 ppm. The presence of succinate linker in the compound **5** was indicated by the multiplet from four protons of two methylene groups in the range 2.58–2.65 ppm on ^1^H NMR spectrum as well as two signals from carbonyl carbons from succinate ester groups at 171.35 and 171.50 ppm on ^13^C NMR spectrum. In both target acylglycerols **4** and **5**, observed multiplicities and chemical shifts for protons from a glycerol skeleton as well as only one signal from carbonyl carbons of ester groups at *sn*-1 and *sn*-3 positions, observed at 173.28 ppm (for acylglycerol **4**) and 173.31 ppm (for acylglycerol **5**), were typical for symmetrical 1,3-diacylglycerols and indicated no acyl migration at the final step of synthesis.

### 2.2. Biophysical Studies

#### 2.2.1. Differential Scanning Calorimetry (DSC)

**Figure 1:**
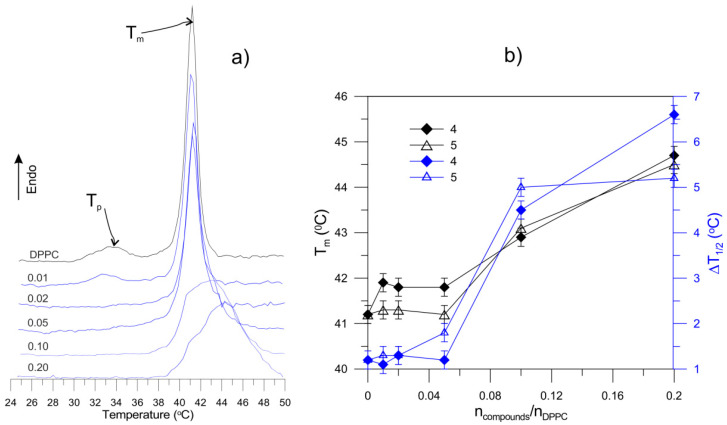
(**a**) Original DSC curves of DPPC and DPPC mixed liposomes with an increasing concentration of acylglycerol **5**; (**b**) The influence of different concentration of acylglycerols of myristic acid containing stigmasterol on the main phase transition temperature T_m_ (black curves) and half-width of the main peak ΔT_1/2_ of DPPC mixed liposomes (blue curves).

Analysis of changes of the physicochemical properties (thermotropic, in particular) of liposomes formed from DPPC and hybrids of acylglycerols of myristic acid with stigmasterol (**4** and **5**), was performed using differential scanning calorimetry (DSC). Figure 1 presents a DSC scan of DPPC and mixed acylglycerol/DPPC multilamellar liposomes for different values of molar ratios of the acylglycerol to DPPC (n_acylglycerol_/n_DPPC_): 0.01, 0.02, 0.05, 0.1, and 0.2. For pure DPPC, literature data regarding the main phase transition temperature, T_m_ (41.2 °C) and the pretransition temperature, T_p_ (35 °C) were confirmed. Incorporation of stigmasterol-containing acylglycerols into liposomes shifts the temperature of the main phase transition towards higher T_m_ values (Figure 1a,b). Similar changes of the main phase transition temperature are caused by both acylglycerols studied. For molar concentrations of 0.1 and 0.2 there is a significant increase of T_m_, which may indicate a decrease of lipid bilayer fluidity in mixed liposomes. As the concentration of compounds **4** and **5** increases, the subtransition disappears and the half-width of the main peak widens considerably. The half width of the main peak is related to the cooperativity of the phase transition. The highest cooperativity was observed for pure DPPC and it significantly decreases with the increased concentration of the tested acylglycerols (Figure 1b). In the case of DPPC, the main transition from the gel phase to the liquid phase is a two-step process. Therefore, the peak is sharp, and the transition occurs at high cooperativity. The addition of new compounds significantly increased the width of the main peak, indicating reduced cooperativity during the phase transition. The increase of the concentration of compounds **4** and **5** in liposomes changes the shapes of the DSC scans. This may additionally indicate the existence of domains within the liposome bilayer. 

#### 2.2.2. Steady-State Fluorescence Spectroscopy

A fluorimetric method was also used to extend the biophysical studies. Using this method, the effect of hybrids of acylglycerols of myristic acid and stigmasterol on the polarity, fluidity, and main phase transition of SUVs liposomes was determined. The following molar ratios of the acylglycerol to DPPC (n_acylglycerol_/n_DPPC_) were used: 0.01, 0.02, 0.05, 0.1, and 0.2. Two fluorescent probes like Laurdan and DPH, which have affinity for two different areas in the membrane, were selected for this study. 

**Figure 2:**
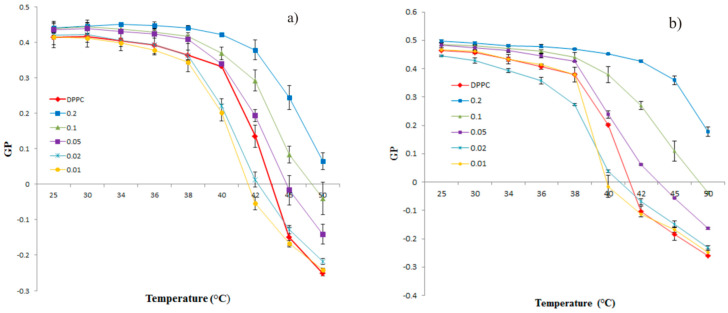
Values of general polarization (GP) of Laurdan in liposomes DPPC (control) and mixed liposomes: acylglycerol **5**/DPPC (**a**) and acylglycerol **4**/DPPC (**b**) in a six-molar ratio of the tested acylglycerol to DPPC (n_acylglycerol_/n_DPPC_): 0.01, 0.02, 0.05, 0.1, and 0.2, as a function of temperature.

Fluorophore of Laurdan takes place near the phospholipid glycerol groups. Because of its location and characteristics, it provides important information about polarity changes in the environment, which is related to the phospholipid phase state. Polarity changes are shown by shifts of the spectrum of emission maximum of Laurdan from 440 nm in gel phase to 490 nm in a liquid crystalline phase. This spectral shift is due to the dipolar relaxation of Laurdan in the lipid environment and is assigned to several water molecules present in the bilayer at the level of the glycerol skeleton [31,32]. Polarization changes were determined using the generalized polarization parameter (GP). Figure 2 presents values of general polarization of Laurdan for liposomes composed of DPPC (control) and mixed liposomes composed of acylglycerol/DPPC at different molar ratios as a function of temperature. The results showed significant differences in GP values in mixed liposomes compared to liposomes formed from pure DPPC. In the case of mixed liposomes, there was a significant increase in GP with an increase of acylglycerol **5** concentration in the range of 0.05–0.2 molar ratio. The differences in GP values started from the lowest temperatures, but the greatest effect was around the temperature of the main phase transition. The opposite effect, i.e., a decrease in GP values relative to the control, was observed for molar ratios of 0.02 and 0.01. These changes started around the pre-phase transition temperature (38 °C) and ended at 45 °C, i.e., already after the phase transition. A higher decrease in GP was observed for the ratio of 0.01. At temperatures above the phase transition, the molar ratio of 0.02 caused a slight increase in GP compared to the control. Liposomes formed with the addition of compound **4** induced similar changes as compound **5**, but with much greater effect. At a molar ratio of 0.2, the presence of compound **4** caused a high increase in GP values in both phases, whereas at molar ratios of 0.02 and 0.01 there was a significant decrease in GP in the gel phase and a small increase in the liquid phase compared to the control. 

An increase in GP value compared to the control indicates an increase of an order in the hydrophilic-hydrophobic region of the membrane (interface of the bilayer), whereas its decrease indicates an increase of a disorder. The results showed that the addition of both acylglycerols in molar ratios ranging from 0.2 to 0.05 causes very strong ordering in the polar heads of the lipid bilayer. The greatest ordering was induced by compound **4** in molar ratio 0.2. On the other hand, at molar ratios of 0.02 and 0.01, both compounds caused an increase in a disorder.

**Figure 3:**
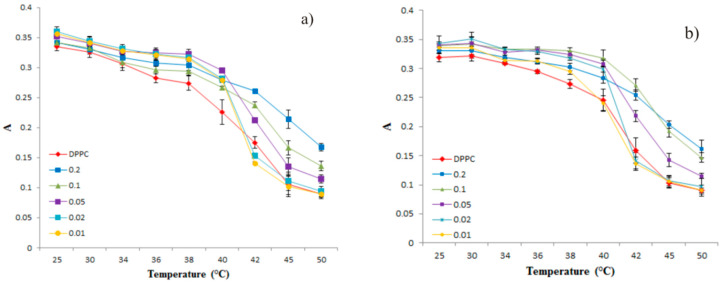
Values of anisotropy of DPH in liposomes DPPC (control) and mixed liposomes: acylglycerol **5**/DPPC (**a**) and acylglycerol **4/**DPPC (**b**) in six molar ratio of the tested acylglycerol to DPPC (n_acylglycerold_/n_DPPC_): 0.01, 0.02, 0.05, 0.1, and 0.2, as a function of temperature.

The second probe used with fluorimetric studies was DPH. This probe has an affinity to be located between the hydrocarbon chains of phospholipids. Because of this localization, information about the main phase transition of lipids and membrane fluidity is obtained, which is related to chain mobility. These shifts are monitored by the change in the fluorescence anisotropy of the DPH probe [33]. An increase in the anisotropy value indicates a decrease in membrane fluidity and thus an increase in the rigidity in the hydrophobic region. A decrease in this value indicates an increase in membrane fluidity, which is associated with an increase in the mobility of the hydrocarbon chains. Figure 3 shows the values of anisotropy of DPH in liposomes formed from DPPC (control) and mixed liposomes composed of acylglycerol **5**/DPPC (a) and acylglycerol **4**/DPPC (b) in six molar ratios as a function of temperature. As with the first probe, the results showed significant differences in values of anisotropy in mixed liposomes compared to liposomes formed from pure DPPC. The trend of changes was similar for both compounds. The results showed an increase in fluorescence anisotropy below and above the temperature of the main phase transition. However, the greatest effect was observed above 40 °C and the changes were proportional to the acylglycerols concentration. Below the main phase transition temperature (40 °C) the effect was opposite, because a greater increase in anisotropy was shown at a molar ratio of 0.02 or 0.01. The obtained results clearly indicated that acylglycerols **4** and **5** cause a significant increase in anisotropy compared to the control, which indicates an increase in the rigidity in the hydrophobic region of the membrane. Moreover, the presence of compounds **4** and **5** in the membranes also shifts the temperature of the main phase transition towards higher temperatures.

## 3. Discussion

In this work we present a new form of acylglycerols containing myristic acid residues at *sn*-1 and *sn*-3 positions and the stigmasterol molecule attached to *sn*-2 position. We used these active substances to form new liposomes in the mixture with model phospholipid and determine their effect on the physicochemical properties of these liposomes.

Using DSC, it has been verified how hybrid acylglycerols of myristic acid with stigmasterol affects the temperature of the main phase transition depending on the molar ratio of the studied compound to DPPC. It was shown that the greatest changes occur for the systems acylglycerol **4**/DPPC and acylglycerol **5**/DPPC at molar ratios 0.1 and 0.2. It was also found that the enthalpy of the main phase transition gradually decreases with increasing acylglycerols **4** and **5**, with the changes being greatest at the molar ratio of acylglycerol/DPPC 0.1 and 0.2. For pure DPPC the enthalpy change is ∆H = 36 ± 1.8 kJmol^−1^, for acylglycerol **4**/DPPC system ∆H = 15.8 ± 0.8 kJmol^−1^ and for acylglycerol **5**/DPPC ∆H = 27.6 ± 1.4 kJmol^−1^, n_acylglycerol_/n_DPPC_ = 0.2. These changes are similar to cholesterol-containing phosphatidylcholine bilayers and plant sterol-containing DPPC bilayers reported in the literature. The same trend of the enthalpy of the main conversion gradually decreasing and the cooperativity of the transition decreasing with increasing sterol concentrations, was observed for acylglycerols **4** and **5** with slight differences among them. A slightly larger effect in both parameters was observed for acylglycerol 4, indicative of a better ability of this compound in lipid disordering a rigid membrane. This resulted in an increase in T_m_ towards higher values, as well as a significant increase in the half-width of the transition. The width of the peak responsible for the main phase transition is closely related to the cooperativity. With a broad, fuzzy peak, a decrease in cooperativity is inferred. A high transition temperature indicates beneficial and stabilizing interactions of sterols with phospholipids [34].

A fluorimetric method was used to determine the effect of hybrid acylglycerols of myristic acid and stigmasterol on the polarity, fluidity and main phase transition of new liposomes. The results obtained using this method confirm the significant effect of both compounds on the hydrophilic and hydrophobic region. Compounds significantly increase the order in the polar heads of the lipid bilayer and cause a decrease in membrane fluidity as well as an increase in the Tm temperature. Moreover, at higher concentrations the phase transition becomes less pronounced and is more extended. These results are consistent with those obtained using the DSC method. Moreover, they may have great significance for their practical application.

An important factor in the practical use of liposomes as carriers of active substances is their stability, which among others is affected by the rigidity of the lipid layer. The regulation of liposome membrane composition (type of lipids, type of sterols and their concentration) can be used as a tool to control the fluidity, permeability, and thermotropic properties of the membrane and thus to predict the release properties, physical, thermal, and oxidative stability. A commercial phospholipid mixture consisting of various natural phospholipids with impurities forms a less homogeneous liposome membrane, which has higher fluidity compared to DPPC [35]. 

A well-studied and frequently used lipid to regulate membrane fluidity is cholesterol. Some of the known properties of this sterol include increasing the packing of phospholipid molecules, decreasing the permeability of the bilayer to non-electrolyte and electrolyte solvents, increasing the resistance of vesicles to aggregation, altering the fluidity of intramolecular interactions so that they become more rigid and persist under high shear stress in the lipid bilayer and decreasing the efficiency of drug incorporation [36,37,38,39,40]. Studies have shown the phospholipid/cholesterol ratio 2:1 as the most suitable combination in terms of characteristics and as the most flexible formulation to release drugs with different physicochemical properties [41].

Plant sterols, despite their beneficial properties (used as cholesterol lowering agents, anti-inflammatory, antibacterial, antifungal, and anticancer agents) have received much less attention as liposome stabilizers compared to cholesterol. Studies comparing cholesterol and plant sterols show their different effects on bilayer permeability, chain melting point, hydrocarbon chain ordering, and on membrane domains [34,42,43]. Wu et al. in their study suggested less efficiency of stigmasterol, compared to cholesterol, in forming ordered domains containing DPPC [34]. However, other studies indicate that phytosterols affect cell membrane properties such as permeability, temperature behavior, structural properties, and stability [44,45]. Silva et al. showed that the β-sitosterol and stigmasterol changed the bilayer ordering and hydration of the polar DPPC head [45]. Jovanović et al. showed that the β-sitosterol decreased the temperature of the main phase transition, reduced membrane fluidity and induced more stable interactions with phospholipids (increased liposome stability) [35]. Unsalan et al. suggested that the incorporation of stigmasterol induces changes in the thermotropic behavior of lipid phases, lipid ordering, fluidity, and hydration near the polar group of liposomes formed from dimyristoylphosphatidylcholine (DMPC) [46]. Similar to our study, their results suggest that stigmasterol is fully able to interact with membranes and alter their biophysical properties. Moreover, these results showed an increase in the ordering of the DMPC membrane in both the gel and liquid phases for low concentrations of stigmasterol (1, 5, and 10 mol%) and a decrease in the ordering in the gel phase as well as a decrease in the conformational disorder of the DMPC membrane in the liquid phase for high concentrations of stigmasterol (20, 30, and 40 mol%). In contrast, our study showed an increase in ordering in the gel and liquid phases for both compounds (**4** and **5**) as well as an increase in the T_m_ temperature, especially for molar ratios acylglycerol/DPPC of 0.1 and 0.2. 

The differences between the literature studies and our results may be due to the structural characteristic of the synthesized stigmasterol derivatives. The aforementioned studies involved a free form of stigmasterol molecule incorporated into a lipid bilayer, where the hydroxy group is directed to the aqueous phase and the hydrophobic rings to the fatty acids of the phospholipids. Our study involved molecules in which stigmasterol is linked to 1,3-dimyristoylglycerol via a carbonate or succinate linker. The hydroxy group of stigmasterol is esterified, which prevents the presence of the molecule in the polar region. In a free stigmasterol, the hydroxy group forms hydrogen bonds with the polar groups of the phospholipid heads. The present study shows that the strong hydrogen bonding caused by stigmasterol on carbonyl and phosphate groups in DMPC membranes suggest that stigmasterol has a significant probability of altering lipid acyl chain flexibility and lipid dynamics.

## 4. Materials and Methods

### 4.1. Chemicals

Dihydroxyacetone (≥98%), stigmasteryl chloroformate, N,N’-dicyclohexylcarbodiimide (DCC, 99%), dimethylaminopyridine (DMP, ≥99%), sodium borohydride (99%), ethanol-free chloroform (≥99%), anhydrous tetrahydrofurane (THF, ≥99.9%), hexane (ACS reagent, ≥99%), 1,2-dipalmitoyl-*sn*-glycero-3-phosphatidylcholine (DPPC, 99%), and sterile filtered water were purchased from Sigma-Aldrich (St. Louis, MO, USA). Other chemicals of analytical grade were purchased from Chempur (Piekary Śląskie, Poland). Stigmasteryl hemisuccinate was synthesized from stigmasterol as described previously [22]. The fluorescent probes, 6-dodecanoyl-2-dimethylaminonaphthalene (Laurdan) and 1,6-diphenyl-1,3,5-hexatriene (DPH), were purchased from Molecular Probes (Eugene, OR, USA).

### 4.2. Analysis

Analytical Thin Layer Chromatography (TLC) was performed on 0.2 mm aluminum plates coated with silica gel 60 F_254_ (Merck). Chromatograms were visualized by spraying the plates with a solution of 1% Ce(SO_4_)_2_ and 2% H_3_[P(Mo_3_O_10_)_4_] in 10% H_2_SO_4_ and heating the plates to 120–200 °C or 0.05% solution of primuline in acetone:water mixture (4:1, *v*/*v*) followed by observation of the spots under UV light (λ = 365 nm).

Flash chromatography was carried out using puriFlash^®^ SX520 Plus Interchim system (Interchim, Montluçon, France) gradient pump, UV detector, and fraction collector. The samples were dry loaded on a pre-column puriFlash^®^ F0012 and the products were separated on puriFlash^®^ SIHP 30 µm columns using gradient elution with mixtures of hexane and ethyl acetate as the mobile phase (flow 26 mL/min, pressure 15 mbar).

Gas chromatography was carried out on an Agilent 6890 N chromatograph using a polyethylene glycol column (DB WAX, 30 m × 0.25 mm × 0.25 µm) and flame ionization detector, with the following temperature parameters: injector 250 °C, detector 280 °C, column: 160 °C (held 3 min), 160–220 °C (rate 5 °C/min), 220–260 °C (rate 30 °C/min), 250 °C (held 3 min). Fatty acid profiles of trimyristin and myristic acid were determined after their conversion to the fatty acid methyl esters (FAME) using BF_3_ × MeOH complex solution according to the standard procedure [47]. Standard FAME mixture (Supelco 37 FAME Mix, Sigma Aldrich, St. Louis, MO, USA) was used as reference.

Spectroscopic measurements in the range of Nuclear Magnetic Resonance (^1^H NMR, ^13^C NMR, COSY, HMQC, HMBC) were recorded on a Jeol 400 MHz Year Hold Magnet spectrometer (Jeol Ltd., Tokyo, Japan) or Bruker Avance II 600 MHz spectrometer (Bruker, Rheinstetten, Germany). Samples were dissolved in CDCl_3_ and the chemical shifts of the detected signals were referenced to the signals of residual solvent (δH = 7.26, δC = 77.00).

Attenuated total reflectance-Fourier transform infrared spectroscopy (ATR-FTIR) spectra were recorded on a Nicolet iS10 spectrometer (Thermo Fisher Scientific, Waltham, MA, USA). 

High Resolution Mass Spectra (HRMS) were registered on Bruker Daltonics ESI-Q-TOF maXis impact mass spectrometer (Bruker, Billerica, MA, USA) using electrospray ionization (ESI) technique.

Boetius apparatus (Nagema, Dresden, Germany) was used to determine melting points (m.p., uncorrected) of synthesized compounds.

### 4.3. Isolation of Trimyristin from Nutmeg

Trimyristin was extracted from nutmeg by Soxhlet extraction using hexane and recrystallized from acetone according to the procedure described in our previous paper [47]. 

### 4.4. Hydrolysis of Trimyristin

Trimyristin (3 g) was dissolved in 50 mL of 1 M NaOH ethanolic solution and the mixture was heated under reflux. After complete hydrolysis of trimyristin (1.5 h, TLC, hexane:diethyl ether 3:1) and cooling the reaction mixture, it was poured into 100 mL of water and acidified with 20 mL of concentrated HCl. Crystals of myristic acid were separated from the solution on a Büchner funnel, washed with 25 mL of distilled water, and dried at 40 °C overnight to afford 2.3 g of myristic acid (yield 81%). 

### 4.5. Synthesis of Target Acylglycerols with Stigmasterol Residues

#### 4.5.1. Preparation of 1,3-Dimyristoylpropanone (**2**)

Solution of myristic acid (1.6 g, 7 mmol) in ethanol-free chloroform was added portion wise to the stirring mixture of dihydroxyacetone (**1**, 0.3 g, 3.3 mmol), DCC (1.44 g, 6.9 mmol), DMAP (0.85 g, 6.9 mmol) and 100 mL of ethanol-free chloroform placed in round-bottom flask. The reaction mixture was stirred at room temperature until the substrate was completely consumed (24 h, TLC: hexane/ethyl acetate 1:1) and filtered using a funnel with G4 sintered disc to remove white precipitate. The filtrate was washed with 0.5 M HCl, neutralized with brine, dried with anhydrous MgSO_4_, filtered, and the solvent was evaporated under vacuum. The crude product was purified by flash chromatography using a gradient elution system (from hexane to hexane:EtOAc 19:1, *v*/*v*). Physical and spectroscopic data of pure 1,3-dimyristoylpropanone **(2**, 1.27 g, yield 75%) are given below:

White crystals, mp 72–75 °C (lit. 76°C [27]), R_f_ = 0.44 (hexane:ethyl acetate, 5:1), ^1^H NMR (400 MHz, CDCl_3_):δ 0.87 (t, *J* = 7.0 Hz, 6H, 2 × -OC(O)(CH_2_)_12_CH_3_), 1.20–1.36 (m, 40H, 2 × -OC(O)CH_2_CH_2_(CH_2_)_10_CH_3_), 1.64 (m, 4H, 2 × -OC(O)CH_2_CH_2_(CH_2_)_10_CH_3_), 2.42 (t, *J* = 7.7 Hz, 4H, 2 × -OC(O)CH_2_(CH_2_)_11_CH_3_), 4.74 (s, 4H, CH_2_-1 and CH_2_-3); ^13^C NMR (100 MHz): δ 14.10 (2 × -OC(O)(CH_2_)_12_CH_3_), 22.67 (2 × -OC(O)(CH_2_)_11_CH_2_CH_3_) 24.81 (2 × -OC(O)CH_2_CH_2_(CH_2_)_10_CH_3_), 29.04, 29.21, 29.34, 29.41, 29.57, 29.62, 29.63 and 29.65 (2 × -OC(O)CH_2_CH_2_(CH_2_)_8_CH_2_CH_2_CH_3_), 31.90 (2 × -OC(O)(CH_2_)_10_CH_2_CH_2_CH_3_) 33.72 (2 × -OC(O)CH_2_(CH_2_)_11_CH_3_), 66.13 (C-1 and C-3), 172.95 (2 × -OC(O)(CH_2_)_12_CH_3_), 198.16 (C-2); IR (ATR, cm^−1^): 1733 (s), 1701 (s), 1162 (s); HRMS (ESI): *m/z* calcd for C_31_H_58_O_5_: 533.4182 [*M*+Na]^+^; found: 533.4179.

#### 4.5.2. Preparation of 1,3-Dimyristoylglycerol (**3**)

To the solution of 1,3-dimyristoylpropanone (**2**, 1 g, 1.2 mmol) in 25 mL of THF placed in an ice bath with NaCl, few portions of NaBH_4_ (0.2 g, 2.6 mmol) were added. When substrate was not detected on TLC plate (30 min, hexane:ethyl acetate, 5:1), THF was evaporated in vacuo, the residues was suspended in water and the product was extracted with CH_2_Cl_2_ (3 × 15 mL). The organic layer was washed with brine until neutral, dried over anhydrous MgSO_4_, filtered and concentrated in vacuo. After flash chromatography (gradient elution from hexane to hexane:ethyl acetate, 5:1, *v/v,*) 0.56 g (yield 56%) of pure 1,3-dimyristoylglycerol (**3**) was isolated. Its physical and spectral data are given below:

White crystals, mp 62–64 °C (lit. 64–66 °C [48]), *R*_f_ = 0.22 (hexane:ethyl acetate, 5:1), ^1^H NMR (400 MHz, CDCl_3_): δ 0.87 (t, *J* = 7.1 Hz, 6H, 2 × -OC(O)(CH_2_)_12_CH_3_), 1.20–1.34 (m, 40H, 2 × -OC(O)CH_2_CH_2_(CH_2_)_10_CH_3_), 1.62 (m, 4H, 2 × -OC(O)CH_2_CH_2_(CH_2_)_10_CH_3_), 2.34 (t, *J* = 7.6 Hz, 4H, 2 × -OC(O)CH_2_(CH_2_)_11_CH_3_), 4.07 (m, 1H, H-2), 4.13 (dd, *J* = 11.3 Hz and 5.6 Hz, 2H, one of CH_2_-1 and one of CH_2_-3), 4.18 (dd, *J* = 11.3 Hz and 4.3 Hz, 2H, one of CH_2_-1 and one of CH_2_-3); ^13^C NMR (100 MHz): δ 14.12 (2 × -OC(O)(CH_2_)_12_CH_3_), 22.68 (2 × -OC(O)(CH_2_)_11_CH_2_CH_3_), 24.86 (2 × -OC(O)CH_2_CH_2_(CH_2_)_10_CH_3_), 29.10, 29.23, 29.34, 29.43, 29.58, 29.63 and 29.66 (2 × -OC(O)CH_2_CH_2_(CH_2_)_8_CH_2_CH_2_CH_3_), 31.90 (2 × -OC(O)(CH_2_)_10_CH_2_CH_2_CH_3_), 34.07 (2 × -OC(O)CH_2_(CH_2_)_11_CH_3_), 65.00 (C-1 and C-3), 68.33 (C-2), 173.95 (2 × -OC(O)(CH_2_)_12_CH_3_); IR (ATR, cm^−1^): 3484 (w), 1715 (s), 1732 (s), 1181 (s); HRMS (ESI): *m/z* calcd for C_31_H_58_O_5_: 535.4338 [*M*+Na]^+^; found: 535.4335.

As a minor product, 0.23 g (23% yield) of *rac*-1,2-dimyristoylglycerol (**3a**) was isolated with the following physical and spectra data: 

White crystals, mp 54–56 °C, (lit. 56–58 °C [49]), *R*_f_ = 0.16 (hexane:ethyl acetate, 5:1), ^1^H NMR (400 MHz, CDCl_3_): δ 0.88 (t, *J* = 7.1 Hz, 6H, 2 × -OC(O)(CH_2_)_12_CH_3_), 1.20–1.34 (m, 40H, 2 × -OC(O)CH_2_CH_2_(CH_2_)_10_CH_3_), 1.62 (m, 4H, 2 × -OC(O)CH_2_CH_2_(CH_2_)_10_CH_3_), 2.32 and 2.34 (two t, *J* = 7.9 Hz, 4H, 2 × -OC(O)CH_2_(CH_2_)_11_CH_3_), 3.71 (dd, *J* = 12.4 Hz and 5.2 Hz, 1H, one of CH_2_-3), 3.74 (dd, *J* = 12.4 and 4.8 Hz, 1H, one of CH_2_-3), 4.23 (dd, *J* = 11.9 and 5.7 Hz, 1H, one of CH_2_-1), 4.32 (dd, *J* = 11.9 and 4.5 Hz, 1H, one of CH_2_-1), 5.08 (m, 1H, H-2); ^13^C NMR (100 MHz): δ 14.12 (2 × -OC(O)(CH_2_)_12_CH_3_), 22.68 (2 × -OC(O)(CH_2_)_11_CH_2_CH_3_), 24.86 and 24.92 (2 × -OC(O)CH_2_CH_2_(CH_2_)_10_CH_3_), 29.07, 29.10, 29.26, 29.35, 29.46, 29.61, 29.64, 29.67 (2 × -OC(O)CH_2_CH_2_(CH_2_)_8_CH_2_CH_2_CH_3_), 31.90 (2 × -OC(O)(CH_2_)_10_CH_2_CH_2_CH_3_), 34.08 and 34.27 (2 × -OC(O)CH_2_(CH_2_)_11_CH_3_), 61.50 (C-3), 61.95 (C-1), 72.04 (C-2), 173.46 (>CHOC(O)(CH_2_)_12_CH_3_), 173.84 (-CH_2_OC(O)(CH_2_)_12_CH_3_); HRMS (ESI): *m/z* calcd for C_31_H_58_O_5_: 535.4338 [*M*+Na]^+^; found: 535.4336.

#### 4.5.3. Preparation of 1,3-Dimyristoyl-2-stigmasterylcarbonoylglycerol (**4**)

1,3-Dimyristoylglycerol (**3**, 0.1 g, 0.19 mmol), stigmasteryl chloroformate (0.32 g, 0.63 mmol)) and DMAP (0.095 g, 0.78 mmol) were dissolved in 50 mL of ethanol-free dry chloroform. The solution was stirred at room temperature for 48 h, and the solvent was evaporated in vacuo. Flash chromatography (gradient elution: hexane to hexane:EtOAc 49:1, *v/v*) afforded 0.17 g (92% yield) of 1,3-dimyristoyl-2-stigmasterylcarbonoylglycerol (**4**) with the following physical and spectral data:

White crystals, mp 43–45 °C, R_f_ = 0.82 (hexane:ethyl acetate, 5:1), ^1^H NMR (600 MHz): δ 0.70 (s, 3H, CH_3_-18s), 0.79 (d, 3H, *J* = 6.5 Hz, CH_3_-27s), 0.80 (d, *J* = 7.4 Hz, CH_3_-29s), 0.84 (d, *J* = 6.4 Hz, 3H, CH_3_-26s), 0.88 (t, *J* = 7.2 Hz, 6H, 2 × -OC(O)(CH_2_)_12_CH_3_), 0.95 (m, 1H, H-9s), 1.00 (m, 1H, H-14s), 1.01 (s, 3H, CH_3_-19s), 1.02 (d, *J* = 6.9 Hz, 3H, CH_3_-21s), 1.06 (m, H-15s (β)), 1.09–1.21 (m, 4H, H-1s (α), H-17s, H-12s (α), one of CH_2_-28s), 1.22–1.33 (m, 41H, 2 × -OC(O)CH_2_CH_2_(CH_2_)_10_CH_3_ and H-16s (β)), 1.38–1.67 (m, 13H, one of CH_2_-28s, H-8s, H-7s (α), CH_2_-11s, H-2s (β), H-24s, H-25s, H-15s (α), 2 × -OC(O)CH_2_CH_2_(CH_2_)_10_CH_3_), 1.70 (m, 1H, H-16s (α)), 1.89 (m, 1H, H-1s (β)), 1.93–2.01 (m, 3H, H-2s (α), H-7s (β), H-12 s (β)), 2.04 (H-20s), 2.31 and 2.32 (two t, *J* = 7.5 Hz, 4H, 2 × -OC(O)CH_2_(CH_2_)_11_CH_3_), 2.36–2.44 (m, 2H, CH_2_-4s), 4.18 and 4.19 (two dd, *J* = 12.0 and 6.0 Hz, 2H, one of CH_2_-1, and one of z CH_2_-3), 4.32 and 4.33 (two dd, *J* = 12.0 and 4.1 Hz, 2H, one of CH_2_-1, and one of CH_2_-3), 4.48 (m, 1H, H-3s), 5.02 (dd, *J* = 15.1 and 8.8 Hz, 1H, H-23s), 5.10 (tt, *J* = 6.0 and 4.1 Hz, 1H, H-2), 5.15 (dd, *J* = 15.1 Hz and 8.7 Hz, 1H, H-22s), 5.39 (m, 1H, H-6s); ^13^C NMR (150 MHz): δ 12.02 (C-18s), 12.24 (C-29s), 14.11 (2 × -OC(O)(CH_2_)_12_CH_3_), 18.96 (C-27s), 19.23 (C-19s), 21.01 (C-11s), 21.08 (C-26s), 21.21 (C-21s), 22.69 (2 × -OC(O)(CH_2_)_11_CH_2_CH_3_), 24.33 (C-15s), 24.82 (2 × -OC(O)CH_2_CH_2_(CH_2_)_10_CH_3_), 25.40 (C-28s), 27.60 (C-2s), 28.89 (C-16s), 29.11, 29.27, 29.36, 29.48, 29.64 29.66 and 29.70 (2 × -OC(O)CH_2_CH_2_(CH_2_)_8_CH_2_CH_2_CH_3_), 31.82 (C-7s), 31.87 (C-8s, C-25s), 31.92 (2 × -OC(O)(CH_2_)_10_CH_2_CH_2_CH_3_), 34.03 (2 × -OC(O)CH_2_(CH_2_)_11_CH_3_), 36.53 (C-10s), 36.81 (C-1s), 37.92 (C-4s), 39.59 (C-12s), 40.48 (C-20s), 42.19 (C-13s), 49.99 (C-9s), 51.23 (C-24s), 55.92 (C-17s), 56.77 (C-14s), 61.99 and 62.00 (C-1, C-3), 72.68 (C-2), 78.46 (C-3s), 123.09 (C-6s), 129.29 (C-23s), 138.28 (C-22s), 139.16 (C-5s), 153.73 (Stig-OC(O)O-), 173.28 (2 × -OC(O)(CH_2_)_12_CH_3_); IR (ATR, cm^−1^): 1739 (s), 1467 (s), 1278 (s), 1254 (s), 1083 (m), 787 (m), 723 (m); HRMS (ESI): *m/z* calcd for C_61_H_106_O_7_: 973.7836 [*M*+Na]^+^; found: 973.7831.

#### 4.5.4. Preparation of 1,3-Dimyristoyl-2-stigmasterylsuccinoylglycerol (**5**)

To the solution of 1,3-dimyristoylglycerol (**3**, 0.21 g, 0.41 mmol), DCC (0.13 g, 0.63 mmol) and DMAP (0.082 g, 0.67 mmol) in ethanol-free chloroform, stigmasteryl hemisuccinate (0.32 g, 0.62 mmol) in chloroform was added dropwise. The reaction mixture was stirred at room temperature for 24 h and worked-up as described in paragraph 4.5.1. Flash chromatography (gradient elution from hexane to hexane:EtOAc 19:1, v/v) afforded 0.308 g (73% yield) of pure 1,3-dimyristoyl-2-stigmasterylsuccinoylglycerol (**5**) with physical and spectral data are given below:

White solid, mp 36–37 °C, R_f_ = 0.57 (hexane:ethyl acetate, 5:1), ^1^H NMR (600 MHz): δ 0.69 (s, 3H, CH_3_-18s), 0.79 (d, 3H, *J* = 6.5 Hz, CH_3_-27s), 0.80 (d, *J* = 7.4 Hz, CH_3_-29s), 0.84 (d, *J* = 6.4 Hz, 3H, CH_3_-26s), 0.88 (t, *J* = 7.2 Hz, 6H, 2 × -OC(O)(CH_2_)_12_CH_3_), 0.95 (m, 1H, H-9s), 1.00 (m, 1H, H-14s), 1.01 (s, 3H, CH_3_-19s), 1.02 (d, *J* = 5.3 Hz, 3H, CH_3_-21s), 1.06 (m, H-15s (β)), 1.10–1.21 (m, 4H, H-1s (α), H-17s, H-12s (α), one of CH_2_-28s), 1.22–1.33 (m, 41H, 2 × -OC(O)CH_2_CH_2_(CH_2_)_10_CH_3_ and H-16s (β)), 1.38–1.63 (m, 13H, one of CH_2_-28s, H-8s, H-7s (α), CH_2_-11s, H-2s (β), H-24s, H-25s, H-15s (α), 2 × -OC(O)CH_2_CH_2_(CH_2_)_10_CH_3_), 1.70 (m, 1H, H-16s (α)), 1.82–1.88 (m, 2H, H-1s (β), H-2s (α)), 1.94–2.01 (m, 2H, H-7s (β), H-12 s (β)), 2.03 (H-20s), 2.30–2.32 (m, CH_2_-4s), 2.31 (t, *J* = 7.6 Hz, 4H, 2 × -OC(O)CH_2_(CH_2_)_11_CH_3_), 2.58–2.65 (m, 4H, (-OC(O)-CH_2_CH_2_-(O)CO-), 4.15 (dd, *J* = 11.9 and 5.9 Hz, 2H, one of CH_2_-1 and one of z CH_2_-3), 4.29 (two dd, *J* = 11.9 and 4.2 Hz, 2H, one of CH_2_-1 and one of CH_2_-3), 4.61 (m, 1H, H-3s), 5.01 (dd, *J* = 15.1 and 8.8 Hz, 1H, H-23s), 5.10 (m, 1H, H-2), 5.15 (dd, *J* = 15.1 Hz and 8.7 Hz, 1H, H-22s), 5.27 (tt, *J* = 5.8 and 4.2 Hz, 1H, H-2), 5.36 (m, 1H, H-6s); ^13^C NMR (150 MHz): δ 12.02 (C-18s), 12.23 (C-29s), 14.11 (2 × -OC(O)(CH_2_)_12_CH_3_), 18.96 (C-27s), 19.28 (C-19s), 21.00 (C-11s), 21.07 (C-26s), 21.20 (C-21s), 22.68 (2 × -OC(O)(CH_2_)_11_CH_2_CH_3_), 24.33 (C-15s), 24.83 (2 × -OC(O)CH_2_CH_2_(CH_2_)_10_CH_3_), 25.39 (C-28s), 27.72 (C-2s), 28.89 (C-16s), 29.12, 29.28, 29.30, 29.36, 29.49, 29.63, 29.66 and 29.69 (2 × -OC(O)CH_2_CH_2_(CH_2_)_8_CH_2_CH_2_CH_3_ and Stig-OC(O)CH_2_CH_2_(O)CO-), 31.82 (C-7s), 31.86 (C-8s, C-25s), 31.91 (2 × -OC(O)(CH_2_)_10_CH_2_CH_2_CH_3_), 34.00 (2 × -OC(O)CH_2_(CH_2_)_11_CH_3_), 36.57 (C-10s), 36.94 (C-1s), 38.04 (C-4s), 39.60 (C-12s), 40.49 (C-20s), 42.18 (C-13s), 50.02 (C-9s), 51.22 (C-24s), 55.91 (C-17s), 56.76 (C-14s), 61.94 (C-1, C-3), 69.44 (C-2), 74.40 (C-3s), 122.72 (C-6s), 129.27 (C-23s), 138.29 (C-22s), 139.50 (C-5s), 171.35 (Stig-OC(O)CH_2_CH_2_(O)CO-), 171.50 (Stig-OC(O)CH_2_CH_2_(O)CO-), 173.31 (2 × -OC(O)(CH_2_)_12_CH_3_); IR (cm^−1^): 1734 (s), 1467 (m), 1154 (s), 1091 (m), 721 (m); HRMS (ESI): *m/z* calcd for C_64_H_110_O_8_: 1029.8098 [*M*+Na]^+^; found: 1029.8085.

### 4.6. Liposome Preparation

Liposomes were prepared using mixture of newly synthesized acylglycerols (**4,5**) and dipalmitoylphosphatidylcholine (DPPC). The procedure was based on the hydration of a dry film and was described in previous papers [50]. Liposomes were formed using different molar ratios of the acylglycerol to DPPC (n_acylglycerol_/n_DPPC_): 0.01, 0.02, 0.05, 0.1, and 0.2. The control liposomes contained only DPPC. The mixture of DPPC and the obtained acylglycerols were dissolved in chloroform. Next, the chloroform was slowly evaporated, first under nitrogen and then under vacuum pump for at least 2 h. Subsequently, the obtained dry film was hydrated with distilled water and shaken intermittently on a vortex at a temperature above the main phase transition of lipids (50 °C). We obtained the multilamellar vesicles (MLVs) when all lipids created a homogeneous milky suspension. Then, small unilamellar liposomes (SUVs) with a diameter of 120–140 nm were formed from MLVs using a Sonics VCX750 sonicator (Sonics, Newtown, CT, USA) for 15 min at 20 kHz. 

### 4.7. Differential Scanning Calorimetry (DSC)

Differential scanning calorimetry method was employed to evaluate the effect of hybrids of acylglycerols with stigmasterol on the thermotropic properties of MLVs liposomes. In particular, the temperature of pre- (Tp) and main phase transition (Tm), as well as the half width of the main peak (⊗T_1/2_) were determined. The final concentration of the lipid in the sample was 25 mg/mL. The method was described precisely in our previous papers [50,51]. Prepared liposomes were incubated for 48 h at 4 °C and measured using Mettler Toledo Thermal Analysis System D.S.C. 821e (Mettler Toledo, LLC, Columbus, OH, USA) in a range temperature from 20 to 55 °C. Measurements were repeated three times and data analysis were performed using original software provided by Mettler Toledo.

### 4.8. Steady-State Fluorescence Spectroscopy

The fluorimetric method was used to determine the changes of polarity, fluidity, and main phase transition of SUVs liposomes composed of stigmasterol-containing acylglycerols and DPPC, prepared according to the procedure described in Section 4.6. The concentration of the lipid in the sample was 0.1 mg/mL. To evaluate the previously mentioned changes, we used two fluorescent probes, namely Laurdan and DPH at concentration 1 µM in the samples. These fluorescent probes were chosen because of their various affinity to the different areas of the membranes. Measurements were conducted above and below the main phase transition of DPPC in a range from 25 to 55 °C using CARY Eclipse fluorimeter (Varian, San Diego, CA, USA) equipped with DBS Peltier temperature controller (temperature accuracy ±0.1 °C). 

The excitation and emission wavelengths were as follows: for DPH, λ_exc_ = 360 nm and λ_em_ = 425 nm; for Laurdan, λ_exc_ = 360 nm and the emission wavelengths were λ_em_ = 440 nm and λ_em_ = 490 nm. The details of this procedure was described in our previous papers [50,51].

## 5. Conclusions

In this work new acylglycerols containing natural myristic acid at *sn*-1 and *sn*-3 positions and stigmasterol residue linked to *sn*-2 position by carbonate and succinate linker were designed, synthesized, and used to the formation of liposomes with DPPC as model phospholipid. The effect of newly synthesized lipids on the physicochemical properties of liposomes was determined, including the polarity, fluidity, and main phase transition using DSC and fluorimetric methods. 

Both stigmasterol-containing acylglycerols in molar ratios ranging from 0.2 to 0.05 significantly increase the order in the polar heads of the lipid bilayer and increase in the rigidity in the hydrophobic region of the membrane. Moreover, the presence of both acylglycerols in the membranes also shifts the temperature of the main phase transition towards higher temperatures. The trend of changes was similar for both compounds and the changes were proportional to the acylglycerols concentration. 

Taking into consideration the beneficial effects of stigmasterol and myristic acid on human health and the stabilization of liposome membranes containing synthesized compounds, the obtained hybrids of acylglycerols and stigmasterol are both good candidates for phytosterols delivery system and more attractive components of liposomes compared to cholesterol. The results of biophysical studies provide a basis for further research on the use of these compounds in the formation of novel lipid nanocarriers.

## Data Availability

The data presented in this study are available on request from the corresponding author.

## Sample Availability

Samples of the compounds are available from the authors.
